# Preoperative prediction of sinonasal papilloma by artificial intelligence using nasal video endoscopy: a retrospective study

**DOI:** 10.1038/s41598-023-38913-0

**Published:** 2023-08-02

**Authors:** Ryosuke Yui, Masahiro Takahashi, Katsuhiko Noda, Kaname Yoshida, Rinko Sakurai, Shinya Ohira, Kazuhiro Omura, Nobuyoshi Otori, Kota Wada, Hiromi Kojima

**Affiliations:** 1 Department of Otorhinolaryngology, Jikei University School of Medicine, Nishi-Shimbashi, Minato-ku, Tokyo Japan; 2 Department of Otolaryngology, Head and Neck Surgery, Toho University Faculty of Medicine, Tokyo, Japan; 3 SIOS Technology Inc., Minami-Azabu, Minato-ku, Tokyo Japan

**Keywords:** Diseases, Pathogenesis

## Abstract

Sinonasal inverted papilloma (IP) is at risk of recurrence and malignancy, and early diagnosis using nasal endoscopy is essential. We thus developed a diagnostic system using artificial intelligence (AI) to identify nasal sinus papilloma. Endoscopic surgery videos of 53 patients undergoing endoscopic sinus surgery were edited to train and evaluate deep neural network models and then a diagnostic system was developed. The correct diagnosis rate based on visual examination by otolaryngologists was also evaluated using the same videos and compared with that of the AI diagnostic system patients. Main outcomes evaluated included the percentage of correct diagnoses compared to AI diagnosis and the correct diagnosis rate for otolaryngologists based on years of practice experience. The diagnostic system had an area under the curve of 0.874, accuracy of 0.843, false positive rate of 0.124, and false negative rate of 0.191. The average correct diagnosis rate among otolaryngologists was 69.4%, indicating that the AI was highly accurate. Evidently, although the number of cases was small, a highly accurate diagnostic system was created. Future studies with larger samples to improve the accuracy of the system and expand the range of diseases that can be detected for more clinical applications are warranted.

## Introduction

Sinonasal inverted papilloma (IP) is a benign tumor that can recur or become malignant, making early diagnosis and surgical resection under endoscopic guidance desirable^1^. Although otorhinolaryngologists use nasal endoscopy for outpatient consultations, there are cases in which it is difficult to distinguish IP from nasal inflammatory polyp. Pathological examination is necessary to make a definitive diagnosis, although it is time-consuming. It would be clinically useful if nasal endoscopy could be used to make a highly accurate supplementary diagnosis. Accordingly, we herein developed a computer-aided diagnosis system to diagnose IP using endoscopic video images.

The recent advances in artificial intelligence (AI) and machine learning technology have provided a foundation for significant applications in the medical field. While hand-written prediction algorithms have long been used to aid medical decision-making, the practical application of machine learning methods for prediction began in 2000. Subsequently, significant improvements in computer hardware performance led to the introduction of DNNs in 2010 and 2012 and the accuracy of DNNs exceeded that of conventional image processing methods at the ImageNet Large Scale Visual Recognition Challenge, eventually surpassing the accuracy of human image recognition in 2015. Nevertheless, large amounts of data are typically required to train DNN models, and their application for the diagnosis of rare diseases, such as IP, is considered challenging.

To the best of our knowledge, no previous study has utilized DNNs to diagnose IP using endoscopic video streams. Therefore, this study aimed to investigate whether DNN models can be used to improve the accuracy of endoscopic diagnosis. Moreover, we compared our DNN models with assessments performed by various otorhinolaryngologists to determine their practicability. Notably, to the best of our knowledge, this is the first study to demonstrate the feasibility of DNN models for the diagnosis of IP using video endoscopy.

## Materials and methods

### Patients

The study protocol was approved by the Human Ethics Review Committee of the Jikei University School of Medicine, Tokyo, Japan (approval number: 32-036 [10111]), which waived the requirement for informed consent owing to the retrospective nature of the study.

We retrospectively evaluated and enrolled 53 patients (male, n = 33; female, n = 30; mean age, 51.2 ± 12.6 years) who underwent endoscopy sinus surgery at our hospital from 2018 to 2021, including 21 patients diagnosed with IP by pathological examination and 32 patients with chronic rhinosinusitis with nasal polyps (CRSwNP). Video images were used to show the nearly bloodless condition prior to manipulation; forceps were not included in the endoscopic image.

### Endoscopy videos

All videos were taken using a rigid 4.0-mm nasal endoscope of 0° angles and a camera head (Olympus Medical System Corp., Japan, and Karl Storz Endoskope, Germany). The main frame rate of the video was 119.88 frames per second, and the resolution was 1920 × 1080 pixels.

### Neural network

We adopted the MobileNet-V2 network, a relatively compact network comprising 88 layers with a fixed input image size of 224 × 244 pixels and 3,538,984 learning parameters.

### Training

The original images were augmented to 6 million images. Augmentation was performed randomly without considering the balance between the number of original images of each patient. During training, the DNN models learned using images resized to 224 × 224 pixels. In each epoch (training cycle), 120,000 images were randomly selected from the aforementioned 6 million images, and a total of 50 epochs were repeatedly performed to train one DNN model. This 50-epoch training procedure was performed with eight datasets, and eight models were generated using one learning set (learning set:evaluation set ratio, 7:1). As DNN models exhibit differences in ability each time they are trained using a large amount of data generated via augmentation from a small number of patients, we created 25 training sets to verify the fluctuations in accuracy of each model. Consequently, 200 models were generated (8 datasets × 25 = 200 models).

### Evaluation

We used square images resized to 224 × 224 pixels. The eight models obtained in each learning set were used as a single evaluation set, and predictions for the 25 evaluation sets were performed as both single-image-unit-based and patient-unit-based predictions. Single-image-unit-based prediction was performed on each single image, whereas patient-unit-based prediction was performed in two ways–continuity analysis and five second (5-s) scoring analysis–with image arrays sequentially aligned according to the order on the original video stream for each patient. In addition to single-model predictions, 25 sets of ensemble predictions combining 24 of the 25 models were used to evaluate the accuracy of the aforementioned predictions. Image-unit-based prediction was simply performed by image-by-image predictions for individual images, whereas patient-unit-based prediction was performed using a continuity analysis and 5-s score analysis for all image sets extracted from a single video of each patient.

#### Continuity analysis

A continuity analysis was one of our original methods to predict if patients were positive or negative for IP. This method initially evaluates the individual images extracted from the video stream individually and subsequently judges whether a patient is positive or negative for IP based on the number of consecutive positive images in the original video stream.

#### Five-second scoring analysis

The 5-s scoring analysis was also an original method for the aforementioned purpose. This method judges whether a patient is positive or negative based on the maximum sum of scores obtained from consecutive images in a 5-s video stream.

### Diagnostic examination by otolaryngologists

All 53 cases were visually diagnosed by 25 otolaryngologists at our hospital. The target videos were the exact same videos used by the AI for training evaluation, unedited at full length. The otolaryngologists included the surgeon; therefore, the primary surgeon for all eligible cases was anonymized. Hence, surgeons were unable to identify the cases they operated on. The percentage of correct diagnoses was compared with that obtained by the AI. As a secondary item, the correct diagnosis rate for otolaryngology was also examined separately by years of practice experience. The skills of the aforementioned 25 otorhinolaryngologists were classified as follows: entry,  < 5 years; intermediate, 4–10 years; and veteran,  > 10 years.

## Results

A total of 143,167 (CRSwNP, n = 57,767; and IP, n = 85,400) images were extracted from 53 endoscopic videos (Table 1). For cross-validation, we randomly divided patients into eight groups and prepared eight datasets using seven groups for learning and the one remaining group for evaluation. Each group was composed such that the number of patients and images was as uniform as possible among all groups. Table 2 shows the number of patients and images in each group. Supplementary Table 1 shows the number of patients and original images in each training set.

**Table 1:** Length of endoscopic videos and number of images for CRSwNP and IP.

Classification	Patients	Total video length (s)	Total images
CRSwNP	32	32.05	57,767
IP	21	47.26	85,400

*CRSwNP* chronic rhinosinusitis with nasal polyps, *IP* inverted papilloma.

**Table 2:** Number of patients with IP and CRSwNP divided into eight groups (A–H) and number of images.

Group	IP		CRSwNP	
	Patients	Images	Patients	Images
A	2	24,687	4	9944
B	2	14,062	4	8176
C	2	9388	4	7071
D	3	8991	4	7086
E	3	7084	4	6719
F	3	7041	4	6350
G	3	7086	4	6314
H	3	7061	4	6107

For cross-validation, we randomly divided patients into eight groups and prepared eight datasets using seven groups for learning and the remaining one group for evaluation. Each group was composed such that the number of patients and images was as uniform as possible.

*CRSwNP* chronic rhinosinusitis with nasal polyps, *IP* inverted papilloma.

### Single-image-unit-based prediction

Supplementary Table 2 shows the sensitivity, specificity, and “average of sensitivity and specificity” (SS-Avg) in single-unit-image-based predictions. Each number shown is the average of 25 single models or 25 ensemble predictions; ensemble prediction had no advantage over single-model prediction in single-image-unit-based prediction.

**Figure 1:**
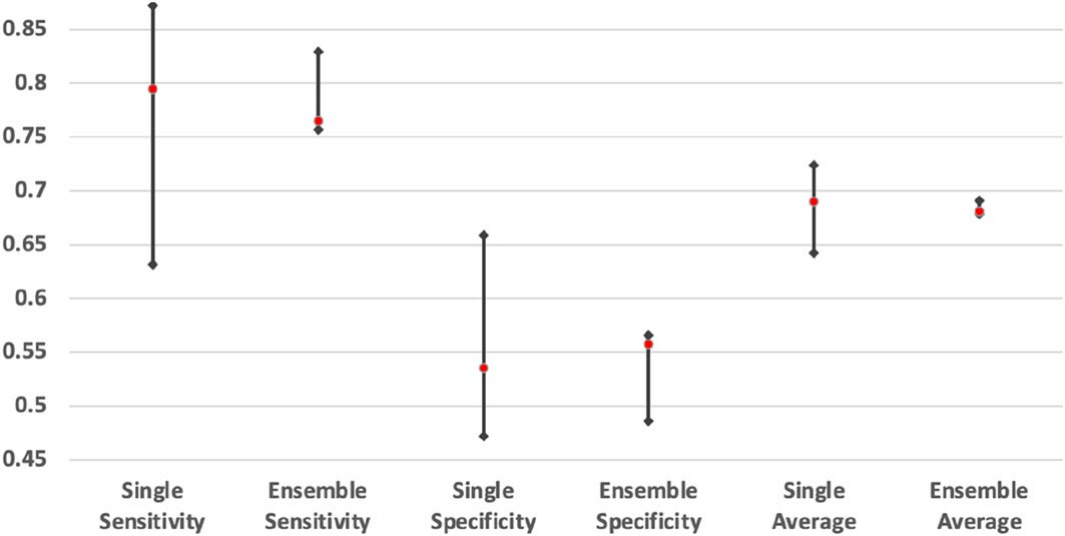
Fluctuation of average accuracy in single-image-based prediction.

The chart in Fig. 1 shows the average fluctuation in accuracy of the single-image-based prediction. There were significant fluctuations between the accuracies of the predictions performed by single models. However, these fluctuations can be minimized by ensemble predictions.

**Figure 2:**
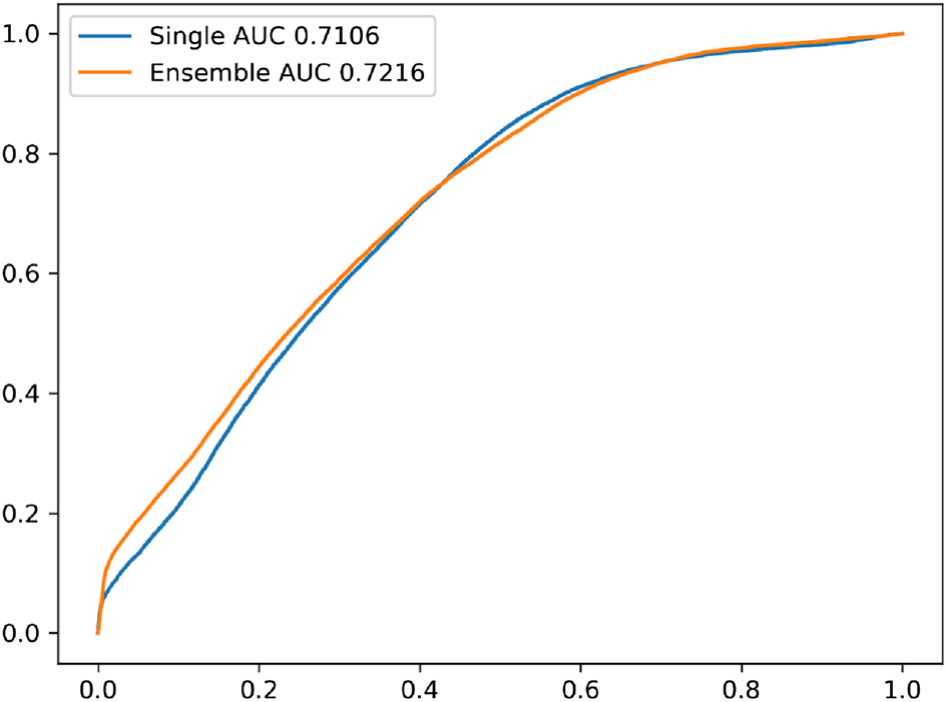
Receiver operator characteristic curve of the media of single-image-based predictions. *AUC* area under the curve.

The chart in Fig. 2 shows the receiver operator characteristic (ROC) curve of the median value of 25 single-model and 25 ensemble predictions in single-image-based prediction. The area under the curve (AUC) of the ensemble prediction was slightly better than that of the single-model prediction.

### Patient-unit-based prediction

Table 3 shows the sensitivity, specificity, and SS-Avg of patient unit-based predictions. The best performance among all patient unit-based predictions was 84.29% (sensitivity, 80.95%; and specificity, 87.63%), as performed by the ensemble prediction using a 5-s score analysis. Each number was the average of 25 single models or 25 ensemble predictions. Ensemble predictions had better performance than single-model predictions, and the 5-s score analysis showed better performance than the continuity analysis.

**Table 3:** Average accuracy of 24 single-model and 24 ensemble predictions in patient-unit-based prediction.

Method	Model	Sensitivity	Specificity	Average
Continuity analysts	Single	77.90%	86.00%	81.95%
	Ensemble	80.95%	87.25%	84.10%
Five-second score analysis	Single	77.52%	86.88%	82.20%
	Ensemble	80.95%	87.63%	84.29%

The best performance in the patient unit-based predictions was 84.29%, as performed by the ensemble prediction using the 5-s score analysis. Ensemble predictions obtained better performance than single-model predictions, whereas the 5-s score analysis obtained better performance than the continuity analysis.

**Figure 3:**
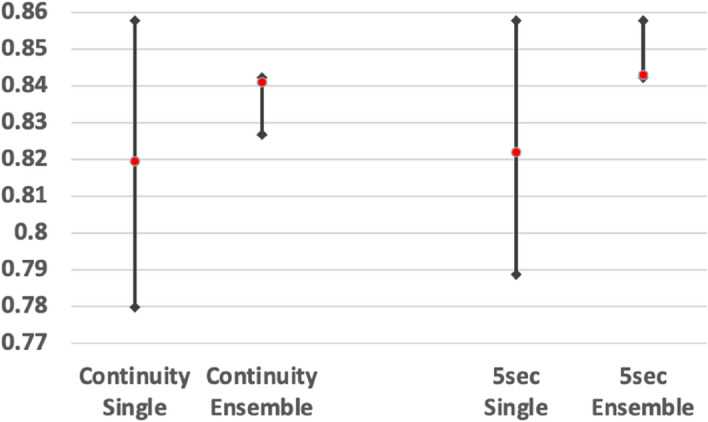
Fluctuation of the average accuracy in single-image-unit-based prediction.

The chart in Fig. 3 shows the fluctuation of the average accuracy in patient-unit-based prediction. The single models demonstrated significant fluctuation, though this can be minimized by ensemble predictions.

### Diagnosis by otolaryngologists

**Figure 4:**
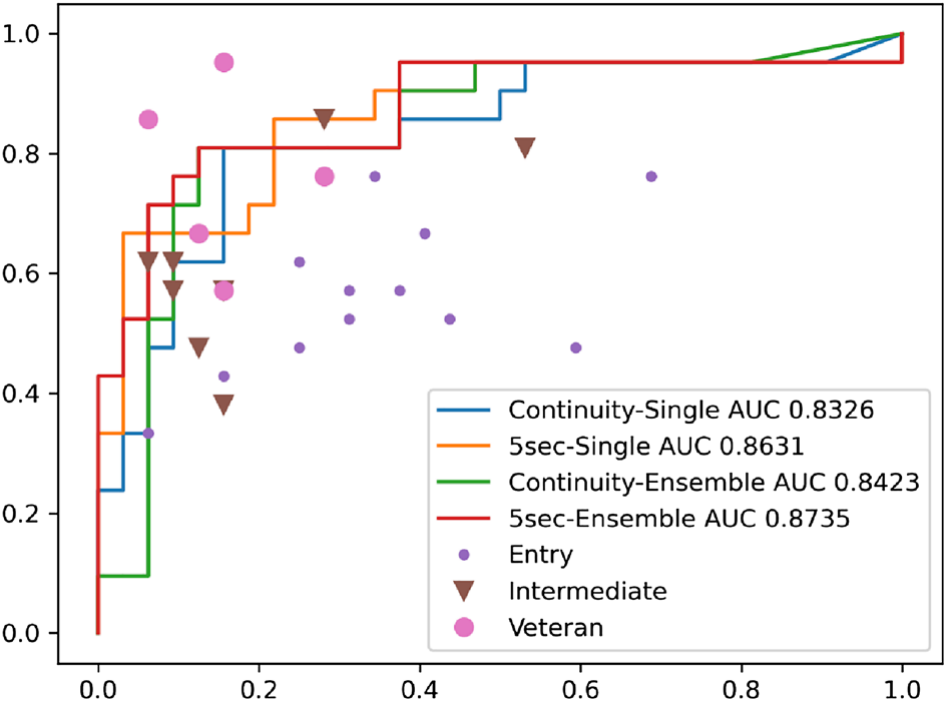
Receiver operator characteristic curve of the median case in patient-unit-based prediction and positive/negative predictive values for 25 otolaryngologists. *AUC* area under the curve.

The chart in Fig. 4 shows the ROC curve of the median of 25 single-model and 25 ensemble predictions in the patient-unit-based prediction model, as well as the positive/negative predictive values plotted for the 25 otolaryngologists. The best AUC was 0.8735, as performed by ensemble predictions using a 5-s peak score analysis. Overall, predictions using the 5-s score analysis obtained better performance than the continuity analysis. The average correct diagnosis rate was 69.4% for all otolaryngologists, 61.6% for the entry group, 74.0% for the intermediate group, and 80.7% for the veteran group. The accuracy of the diagnoses of otolaryngologists tended to increase with each grade level. All AI diagnostic systems outperformed more than half of the otolaryngologists and were as accurate as the veteran group.

## Discussion

Generally, large amounts of data are typically required to train DNN models, and their application in diagnosing rare diseases remains challenging. Therefore, developing disease diagnostic methods that improve accuracy with a small number of samples is essential for medical AI research. Nevertheless, the AI studies we performed, one of which used hysteroscopic videos and another predicted the extent of middle ear cholesteatoma development, demonstrated high accuracy with a small sample size^2,3^.

IP occurs in approximately 1.5 cases per 100,000 people annually^4^, recurs in approximately 15% of cases, and becomes malignant in approximately 5% of cases^1^. IP may have a characteristic “raspberry-like” appearance, but it is often difficult to determine the cause of recurrence^5^. A model that can distinguish IP from squamous cell carcinoma with an accuracy of 77.9% using magnetic resonance imaging has been reported^6^. In any case, a definitive diagnosis is made by pathology, although it is time-consuming. Girdler constructed a system to diagnose IP using simple images, with an accuracy of 74.2%^7^. Our study is the first clinical study using nasal endoscopic video, which allowed us to develop a computer-aided diagnosis model that is more accurate than previous models.

**Figure 5:**
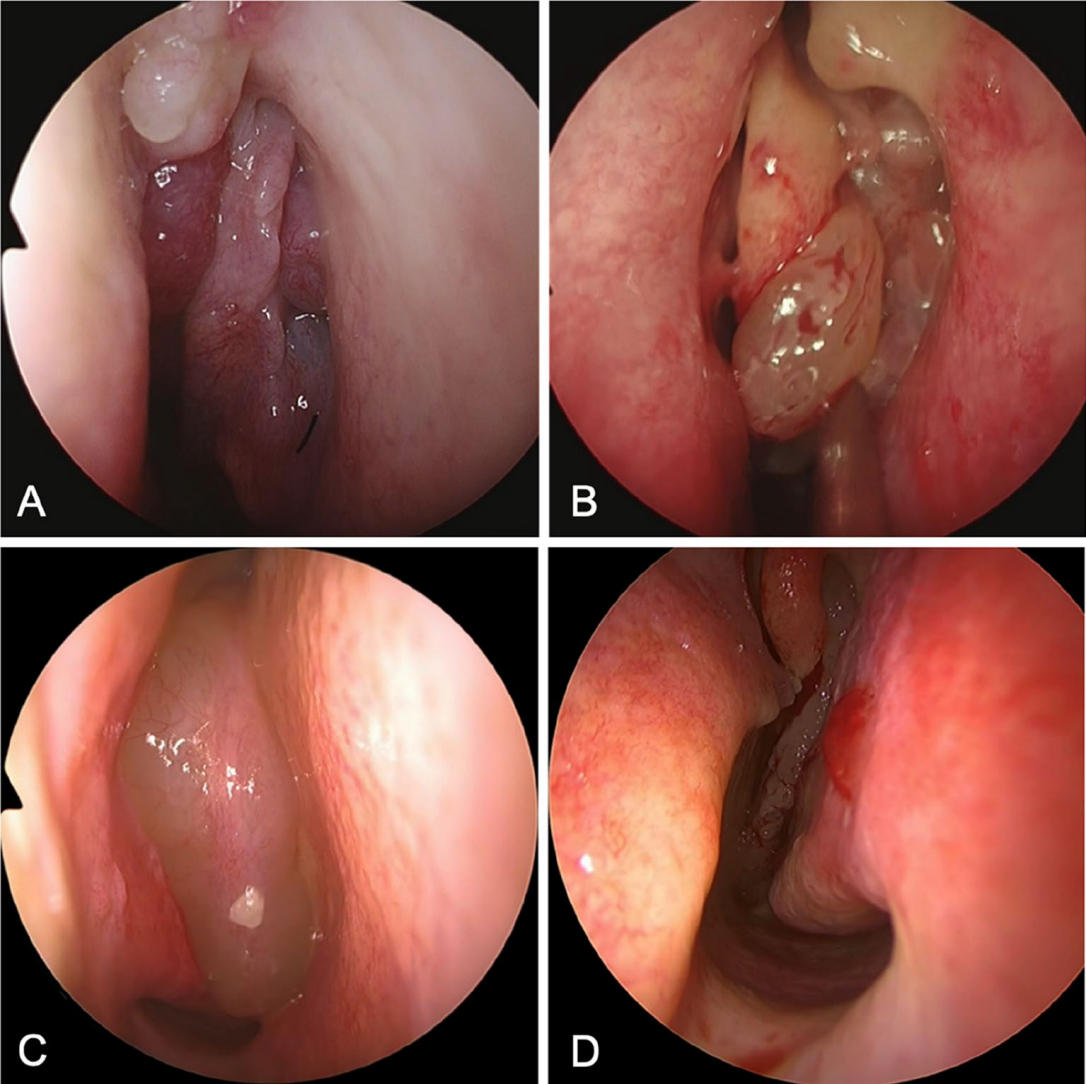
(**A**,**B**) Endoscopic images of cases with a low human (otorhinolaryngologists) correct response rate but high AI correct response rate ((**A**) [CRSwNP]: otorhinolaryngologists, 28% and AI, 100%; (**B**) (IP): otorhinolaryngologists, 12% and AI, 100%). (**C**,**D**) Endoscopic images of cases with a low AI correct response rate but high human correct response rate ((**C**) [CRSwNP]: otorhinolaryngologists, 96%; and AI, 0.96%; (**D**) (IP): otorhinolaryngologists, 76%; AI, 0%). *CRSwNP* chronic rhinosinusitis with nasal polyps, *IP* inverted papilloma, *AI* artificial intelligence.

The accuracy of the AI models was higher than the correct diagnosis rate of otolaryngologists. To explain this phenomenon, we examined cases in which AI and otolaryngologists differed in accuracy. Initially, there was a case in which AI was hardly able to obtain any correct findings indicative of IP, although they were obvious to the otolaryngologist (Fig. 5). Although there may be findings in the images that were missed by the otolaryngologist, this may be because that the machine-trained cases did not contain similar findings, since the AI cannot guarantee sufficient performance in evaluating new images when it has learned many images in the same lineage^8^. Moreover, AI tends to be weak in detecting distant lesions in images^9^ and may not have been able to recognize fine mucosal changes. Second, there were cases in which the correct diagnosis rate by otolaryngologists was low, though the AI correctly diagnosed all cases (Fig. 5). We believe that this is the case because AI recognizes different areas from humans; nevertheless, generally, the specific judgment criteria of AI are unknown and called a black box. There is a possibility that the key to detecting lesions is hidden in these cases.

This study has some limitations. First, the number of cases was small. Second, the edited endoscopic surgical videos were clipped from scenes without any manipulation with bleeding, but forceps and bleeding are slightly seen in the video. Therefore, it may be difficult to say that the information is purely about mucosal lesions. To solve this problem, it will be necessary in the future to image the lesion for a longer period of time preoperatively. While for DNN the cases used for learning were separated from those used for evaluation, the otolaryngologists used all cases for evaluation; therefore, the evaluation criteria are not precisely identical. Hence, it is inappropriate to compare the two. By presenting the diagnostic accuracy of IP for general otolaryngologists, we consider this as one indicator to evaluate DNN accuracy. Further improvement of accuracy is essential for future clinical application. Multicenter clinical research is also warranted. We are also planning to increase the number of diseases covered, and the ability to screen various diseases using a nasal endoscopy will enable the use of AI in health checkups and non-specialist consultations.

## Conclusion

We were able to develop an AI diagnostic system that diagnoses IP with a relatively high accuracy using video, although the number of cases was small. Future studies with more cases and diseases to build a more accurate and practical diagnostic aid system are warranted.

## Supplementary Information

Supplementary Tables.

## Data Availability

Data available: Yes. Data types: De-identified participant data. How to access data: takahashima@jikei.ac.jp. When available: Upon publication.
