# Supplementary material for: Preoperative prediction of sinonasal papilloma by artificial intelligence using nasal video endoscopy: a retrospective study
Source: Sci Rep. 2023 Aug 2;13:12439. doi: 10.1038/s41598-023-38913-0 (PMC10397257; doi:10.1038/s41598-023-38913-0)
Supplement: Supplementary file 1 — Supplementary Tables. [file 41598_2023_38913_MOESM1_ESM.docx]

**Preoperative prediction of sinonasal papilloma by artificial intelligence using nasal video endoscopy: A retrospective study**

Ryosuke Yui, Masahiro Takahashi, Katsuhiko Noda, Kaname Yoshida, Rinko Sakurai, Shinya Ohira, Kazuhiro Omura, Nobuyoshi Otori, Kota Wada, Hiromi Kojima

Supplements

Supplementary Table S1 Number of patients and images used for the training and evaluation of eight training sets

| Training Set | Training | | | | | Evaluation | | | | |
| --- | --- | --- | --- | --- | --- | --- | --- | --- | --- | --- |
|  | Group | IP | | CRSwNP | | Group | IP | | CRSwNP | |
|  |  | Pat | Img | Pat | Img |  | Pat | Img | Pat | Img |
| Set 1 | A, B, C, D, E, F, and G | 19 | 60713 | 28 | 47823 | A | 2 | 24687 | 4 | 9944 |
| Set 2 | B, C, D, E, F, G, and H | 19 | 71338 | 28 | 49591 | B | 2 | 14062 | 4 | 8176 |
| Set 3 | C, D, E, F, G, H, and A | 19 | 76012 | 28 | 50696 | C | 2 | 9388 | 4 | 7071 |
| Set 4 | D, E, F, G, H, A, and B | 18 | 76409 | 28 | 50681 | D | 3 | 8991 | 4 | 7086 |
| Set 5 | E, F, G, H, A, B, and C | 18 | 78316 | 28 | 51048 | E | 3 | 7084 | 4 | 6719 |
| Set 6 | F, G, H, A, B, C, and D | 18 | 78359 | 28 | 51417 | F | 3 | 7041 | 4 | 6350 |
| Set 7 | G, H, A, B, C, D, and E | 18 | 78314 | 28 | 51453 | G | 3 | 7086 | 4 | 6314 |
| Set 8 | H, A, B, C, D, E, and F | 18 | 78339 | 28 | 51660 | H | 3 | 7061 | 4 | 6107 |

CRSwNP, chronic rhinosinusitis with nasal polyps; IP, inverted papilloma; Pat, patients; and Img, images

Supplementary Table S2 Average accuracy of 25 single-model and 25 ensemble predictions in single-image-based prediction

| **Model** | **Sensitivity** | **Specificity** | **Average** |
| --- | --- | --- | --- |
| Single | 79.46% | 53.56% | 69.01% |
| Ensemble | 76.45% | 55.78% | 68.11% |

Ensemble prediction has no advantage over single-model prediction in a single-image-unit-based prediction.
